# Can thermostable vaccines help address cold-chain challenges? Results from stakeholder interviews in six low- and middle-income countries

**DOI:** 10.1016/j.vaccine.2016.01.001

**Published:** 2016-02-10

**Authors:** Debra D. Kristensen, Tina Lorenson, Kate Bartholomew, Shirley Villadiego

**Affiliations:** PATH, PO Box 900922, Seattle, WA 98121, USA

**Keywords:** Cold chain, Vaccine stability, Thermostable, Controlled-temperature chain

## Abstract

**Introduction:**

This study captures the perspectives of stakeholders at multiple levels of the vaccine supply chain regarding their assessment of challenges with storing vaccines within recommended temperature ranges and their perceptions on the benefits of having vaccines with improved stability, including the potential short-term storage and transport of vaccines in a controlled-temperature chain.

**Methods:**

Semi-structured interviews were undertaken with 158 immunization stakeholders in six countries. Interviewees included national decision-makers and advisors involved in vaccine purchasing decisions, national Expanded Programme on Immunization managers, and health and logistics personnel at national, subnational, and health facility levels.

**Results:**

Challenges with both heat and freeze-exposure of vaccines were recognized in all countries, with heat-exposure being a greater concern. Conditions leading to freeze-exposure including ice build-up due to poor refrigerator performance and improper icepack conditioning were reported by 53% and 28% of participants, respectively. Respondents were interested in vaccine products with improved heat/freeze-stability characteristics. The majority of those involved in vaccine purchasing indicated they would be willing to pay a US$0.05 premium per dose for a freeze-stable pentavalent vaccine (68%) or a heat-stable rotavirus vaccine (59%), although most (53%) preferred not to pay the premium for a heat-stable pentavalent vaccine if the increased stability required changing from a liquid to a lyophilized product. Most respondents (73%) were also interested in vaccines labeled for short-term use in a controlled-temperature chain. The majority (115/158) recognized the flexibility this would provide during outreach or should cold-chain breaks occur. Respondents were also aware that possible confusion might arise and additional training would be required if handling conditions were changed for some, but not all vaccines.

**Conclusion:**

Participating immunization stakeholders recognized the benefits of vaccine products with improved stability characteristics and of labeling vaccines for controlled-temperature chain use as a means to help address cold-chain issues in their immunization programs.

## Highlights

• Interviews (158) conducted with immunization personnel in 6 countries.
• Respondents are interested in vaccines with improved heat and freeze stability.
• Most involved in vaccine purchases would pay a slight premium for those features.
• Most saw value in controlled temperature chain (CTC) use of vaccines.
• Many highlighted the need for careful consideration of CTC risks/benefits.

## 1 Introduction

The challenge of transporting and storing vaccines at refrigerated temperatures (2–8 °C) is being addressed on many fronts. The need to address this challenge has become increasingly important because of the introduction of new and more expensive vaccines that are at risk of damage from heat and/or freeze exposure [1], [2], [3], [4] and that cumulatively may overwhelm the already insufficient cold-chain capacities of many countries [5], [6], [7], [8].

Efforts to increase the heat and freeze stability of some vaccine products have been quite successful [9], and in 2012 the World Health Organization (WHO) proposed new programmatic-suitability requirements for vaccines purchased by United Nations agencies that set mandatory minimum stability standards and signaled a preference for vaccines that are heat- and freeze-stable and that can be stored for extended periods of time above 8 °C [10]. In 2013, MenAfriVac^®^ became the first WHO-prequalified vaccine labeled for controlled-temperature chain (CTC) use, allowing a single excursion of up to four days at 40 °C [11], [12]. Over the years, a number of countries have piloted or adopted protocols permitting several vaccines to be kept at ambient temperatures for a defined period of time to ease logistical challenges and eliminate the need for icepacks for transport [13], [14], [15], [16], [17].

To our knowledge, no study to date has captured stakeholder perspectives at multiple levels of the vaccine supply chain regarding the need for vaccines with improved stability and their opinions on how use of vaccines in a CTC might help to mitigate supply chain challenges. This article summarizes the views of stakeholders in six countries of varying income classifications that have recently introduced new vaccines into their national immunization programs. The results are meant to inform ongoing initiatives to improve vaccine distribution and storage through enhanced vaccine stability.

## 2 Methods

The opinions of 158 immunization stakeholders in Brazil, China, India, Peru, the Philippines, and Tanzania (both mainland and Zanzibar) were sought via semi-structured, one-on-one interviews between October 2011 and March 2012. Countries were selected to represent demographic, geographic, and economic diversity. Interview sites and participants were chosen in collaboration with the Expanded Programme on Immunization (EPI) manager (or equivalent) in each country using a sampling methodology inclusive of all levels of the supply chain and representative of low and high rates of third dose of diphtheria, tetanus, pertussis (DTP3) combination vaccine coverage. A summary of study participants is presented in Table 1.

Three different semi-structured questionnaires were used for the interviews, tailored to the roles of the interviewees as follows: (1) national decision-makers and advisors involved in vaccine-purchasing decisions, (2) national EPI managers, and (3) health and logistics personnel, including subnational EPI managers, logisticians, physicians, and nurses. The questionnaires were developed in English, translated into local language where necessary, and pilot-tested. A local immunization expert and a translator, if needed, were present at the time of the interview to ensure questions and responses were clearly articulated and accurately transcribed.

Survey questions reported on in this article focused on cold-chain challenges, the value of more stable vaccines, and the CTC concept.^2^ Survey responses were translated (if needed), aggregated, and analyzed in Excel.

## 3 Results

### 3.1 Perceptions and challenges with vaccine heat and freeze exposure

**Fig. 1:**
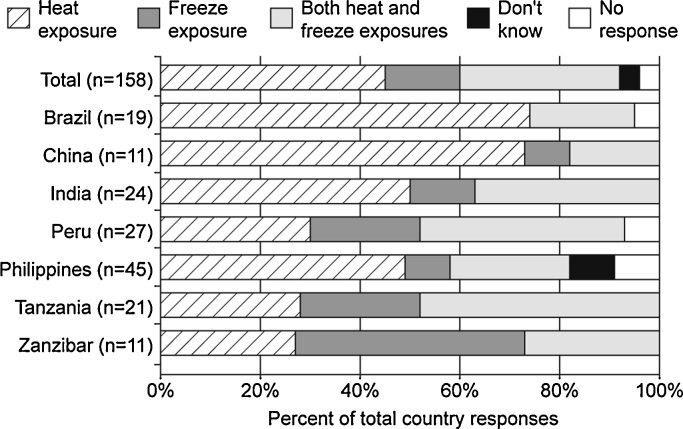
Perceived concerns regarding vaccine temperature exposures reported by country.

To better understand the perceived need for vaccines with heat and freeze stability, participants were asked whether exposure to heat or freezing temperatures was of greater or equal concern. Forty-six percent of all participants (73/158) believed that exposure to heat was a greater concern, 32% (50/158) believed that heat and freezing were equal concerns, 15% (24/158) believed freeze-exposure to be the primary concern, 3% (4/158) reported that they did not know, and 4% (7/158) did not respond. Fig. 1 details the responses by country.

**Fig. 2:**
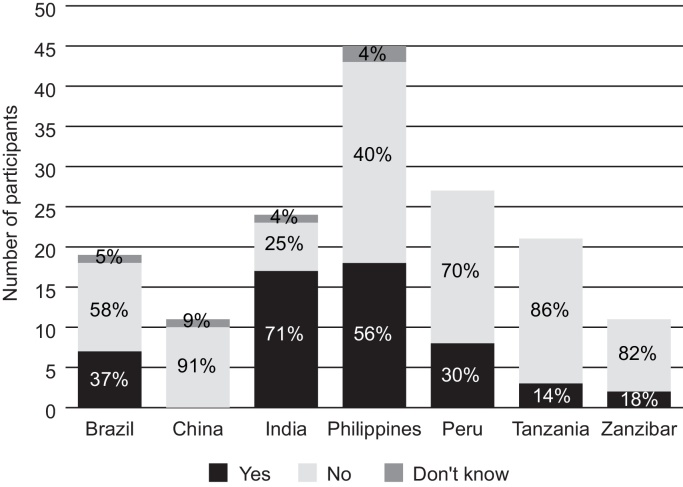
Participants stating that exposure of vaccines to freezing temperatures occurs in their immunization program.

When asked specifically whether exposure of vaccines to freezing temperatures is a problem in their immunization program, only 35% (55/158) of participants said “yes.” Fig. 2 shows the breakdown by country. To better understand the likelihood of exposure to freezing, the following questions were then asked: “Are icepacks typically taken directly from the freezer and placed into the vaccine carrier prior to outreach (i.e., without conditioning)?” and “Does ice build-up occur inside the refrigerator or do temperatures fall below 0 °C in the refrigerator?” Sixty-four percent (101/158) of respondents said “yes” to one or both of these questions indicating awareness of conditions that could lead to freezing.

**Fig. 3:**
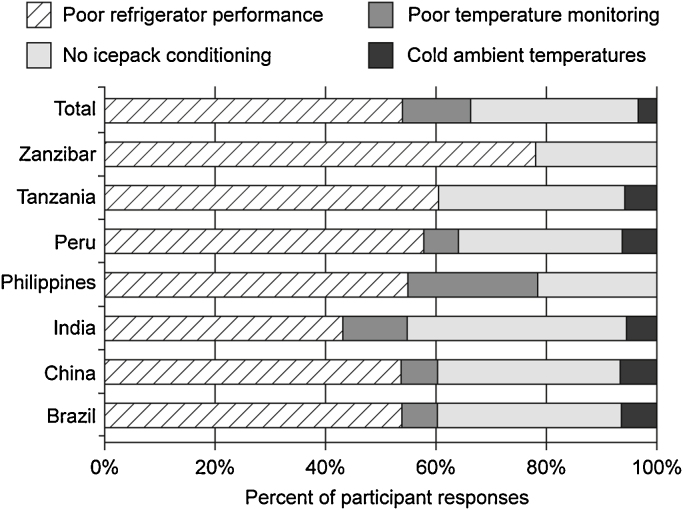
Potential sources of vaccine freeze-exposure reported by country.

Poor refrigerator performance was reported by 53% (84/158) of participants and improper icepack conditioning was reported by 28% (44/158) of participants (Fig. 3).

### 3.2 Reported incidence of vaccine wastage

Thirty-five percent (56/158) of participants recalled one or more incidents over the last few years resulting in the discard of large quantities of vaccine. Forty-four percent (70/158) believed that no incidents had occurred, 2% (3/158) did not know, and 18% (29/158) did not respond. Among the reasons given for loss of a large quantity of vaccine, the majority (29/60) was due to expiry, followed by heat exposure (22/60). Some of the incidents leading to heat exposure included power outages, equipment failures, transportation delays, or delivery truck arrival on a holiday or after working hours.

### 3.3 Opinions on use of a controlled-temperature chain

Participants were provided with the following scenario before being asked a series of questions about potential use of a CTC for vaccines:

It might be possible for some vaccines to be labeled as follows: “Store from +2 °C to +8 °C. Can be stored up to 37 °C for 30 days or less. Do not freeze.”^3^

Overall, 73% (115/158) of participants believed there would be circumstances where it might be helpful to temporarily store or transport vaccines labeled as above without refrigeration or ice. Across all countries, 85% (33/39) of participants at the facility, 74% (37/50) at the municipal/district, 71% (20/28) at the regional/provincial, and 61% (25/41) at the national level believed it would be helpful. By country, the proportion of participants who thought CTC use would be helpful were as follows: mainland Tanzania (95%), Philippines (87%), Brazil (79%), India (75%), Zanzibar (64%), Peru (44%), and China (36%).

All participants were asked to imagine circumstances in their immunization program where having vaccines labeled for CTC use could be beneficial. Open-ended responses included: during power outages or when gas or backup generators are not available (35 responses), at peripheral health facilities without refrigerators (29 responses), to facilitate outreach (22 responses), to provide an additional assurance of heat stability when means to monitor cold chain equipment is difficult or unavailable (19 responses), and when ice is not available or ice in vaccine carriers melts (18 responses). Five participants mentioned that this strategy should be used only in a campaign setting and not in routine immunization due to the potential for confusion.

All participants were asked their opinion on the minimum length of time for vaccine storage or transport without ice or refrigeration that they believed would be useful, and 84% (132/158) gave their opinion while 26 participants did not know or said no amount of time would be useful. The responses have been clustered in time ranges for brevity. Less than 1 day was proposed by 16% (21/132), 1–2 days by 11% (14/132), 3–7 days by 13% (17/132), 8–14 days by 9% (12/132), 15–30 days by 34% (45/132), and over 30 days by the remaining 17% (23/132) of respondents. The responses from those suggesting shorter periods (2 weeks or less) of CTC use generally corresponded to interest in using a CTC when cold-chain breaks occur or to facilitate outreach or short-term storage at remote facilities lacking access to power. The suggested longer time periods (greater than 3 months) for CTC use generally reflected interest in routine storage of all vaccines without refrigeration.

Of the 27% (43/158) who did not perceive any benefits from CTC use for vaccines, 91% (39/43) expressed concern that it would be too confusing and pose a safety risk if some but not all vaccines could be used in a CTC. In addition, 28% (12/43) considered that health workers would not be able to ensure vaccine quality during CTC use, especially in climates with temperatures exceeding 37 °C. The new WHO threshold of 40 °C might help to mitigate some of this concern.

### 3.4 Opinions about tradeoffs between vaccine product cost, presentation format, and improved stability

National decision-makers, advisors, and EPI managers (*n* = 36) were asked three theoretical questions to determine their preferences with regard to alternative vaccine products. A representative price increase of US$0.05 per dose was chosen, which does not indicate actual or predicted price increases. Sixty-eight percent (19/28) of those who responded stated they would be willing to pay more for a liquid pentavalent vaccine that could not be damaged by freezing, with remarks such as “a freeze stable vaccine would be more cost-effective,” it would “offer greater safety,” it is “worth the price because it will provide more efficacy and protection,” and it “would be easier to manage vaccines; there would be less need for monitoring and training on the shake test.”

Twenty-five percent (7/28) would prefer to purchase pentavalent vaccine at the current price, even though it is susceptible to freeze damage. Participants from Brazil, the Philippines, Tanzania, and Zanzibar who chose the current pentavalent vaccine commented that there was not a problem with freezing of vaccines in their country. A participant from India commented that, “with the millions of vaccines purchased annually, 1 rupee per dose would result in a large increase in price.” Two participants responded that they did not know. One participant from the Philippines commented “We have to yet discuss the pros and cons, estimate the costs and compare, address operational issues.”

Fifty-nine percent (19/32) of the responding participants would be willing to pay more for a liquid rotavirus vaccine that is heat stable. Of these, six commented that a more stable vaccine would facilitate reductions in transport and storage costs and vaccine wastage. A participant from Brazil commented, “This would [be] an interesting study for finance to perform a risk-benefit analysis.” A participant from Tanzania commented, “For all vaccines, I would always go for the most stable, because this reduces the risk.”

In contrast, 34% (11/32) would prefer the current rotavirus vaccine that is less heat stable at the current price. One participant commented that the “cost is not justifiable in Peru, as [the country] does not have a policy to take vaccines out of the cold chain.” Two participants remarked that price is important and 7 days of heat stability is sufficient. Two participants did not express a preference.

When compared to a lyophilized hepatitis vaccine that is more heat stable at the same price, 53% (17/32) of respondents would prefer a hepatitis B vaccine that is liquid with the stability of current products. Five participants commented that lyophilized vaccines should be avoided when possible, as they take time, can cause confusion, and require reconstitution syringes and diluents that are often kept outside of the refrigerator until the day of vaccination. One participant commented, “The concern is at the lower level – if they can keep [a vaccine] for one month without a refrigerator, that would be worthwhile. But, if the refrigerator is already there, this does not offset the cost of the time spent to reconstitute, store diluent, syringe, etc.”

Forty-one percent of those responding (13/32) preferred a hepatitis B vaccine that is lyophilized and more heat stable, noting that a more stable product would “reduce risk,” “reduce cost,” and “reduce wastage.” One participant commented “[we] could keep the vaccine for a long time for birth-dose delivery.”

## 4 Discussion

While previous studies have assessed vaccine cold chains [18], [19], monitored temperatures during vaccine storage and distribution [20], [21], and focused on the use of specific antigens in a CTC [22], [23], [24], our research attempts to offset the paucity of data on the perceptions of country stakeholders regarding their challenges in maintaining vaccines in a 2–8 °C temperature range and interest in purchasing and using vaccine products with improved stability.

### 4.1 Heat exposure, heat-stable vaccines, and CTC use of vaccines

It is important to note that this survey captured the perceived causes of vaccine loss and not the actual amounts of vaccine lost due to expiry or to heat or freeze damage. In this context, discards due to expiration ranked first, heat exposure ranked second, and freeze exposure ranked third. The evidence base of actual vaccine wastage due to heat and freeze exposure remains sparse. Three of the six countries surveyed in this study (Brazil, China, and Peru) do not have vaccine vial monitors to help identify when vaccines have received excessive heat exposure and should be discarded, or whether they can be retained after cold chain breaks. Interestingly, Brazil and China had the greatest percentage of participants expressing concerns about vaccine heat exposure, possibly because vaccines are discarded as a precaution when heat exposures occur. Additional research would be required to see whether this is the case.

There are several ways to prevent vaccine wastage from heat exposure, including improved heat stability to prolong the useful life of vaccines when they are exposed to higher temperatures. While our sample size of vaccine purchasing decision-makers and advisors is small (36 national level respondents), the results from this study indicate that the majority (59%) would be willing to pay more for a liquid rotavirus vaccine that is more heat stable. However, a slight majority (53%) would be unwilling to switch to a more heat-stable hepatitis B vaccine at the same price if the switch meant moving from a liquid to a lyophilized product. Lyophilized vaccines require diluent and must be reconstituted before use, resulting in increased product volumes, logistical concerns, and potential safety issues.

Vaccines can be labeled for higher temperature storage if vaccine manufacturers have conducted sufficient stability testing and obtained regulatory approval. Efforts continue to assess the pros and cons of intentionally removing key heat-stable vaccines from the cold chain at the periphery of health systems to facilitate outreach and improve immunization coverage [11], [12], [23]. Whenever these vaccines are exposed to ambient temperatures, care must be taken to ensure that the exposure temperature does not exceed the limit on the label (e.g., 40 °C for not more than 4 days for MenAfriVac).

The majority of interviewees felt that such use of a CTC for vaccines would be feasible and beneficial for making longer outreach trips and that more heat stable vaccines would provide some allowable margin for temporary heat excursions during cold-chain breaks. Twenty-two percent (29/132) thought that the minimum length of time needed for keeping vaccines in a CTC should be between 3 days and 2 weeks – an achievable target for many vaccines that also would be compliant with new WHO guidelines [25]. Use of a CTC for vaccines may offer other advantages that participants also acknowledged, such as the avoidance of vaccine freezing, removal of the need to freeze and condition icepacks, and less weight for transport when ice is not used.

Respondents were aware of the complexity of having different rules for different vaccines and the accompanying need for additional training and tools to appropriately implement vaccine CTC use [12], [26]. Further CTC studies are needed to advance the understanding of the feasibility, costs, and benefits of intentional removal of vaccines from cold chains. Ideally, more vaccines will be labeled for higher temperature storage in the future in accordance with WHO's existing recommendation [10].

### 4.2 Freeze exposure and freeze-stable vaccines

Exposure to freezing temperatures was recognized as a concern in all countries by at least a few participants, though it was reported to be a lesser cause of vaccine loss than expiry and heat exposure. However, conditions leading to vaccine freeze exposure were identified by the majority of participants, suggesting that vaccine freezing may occur, but goes unrecognized. In a review of cold-chain monitoring studies in developing countries, 35% of shipments and 22% of storage facilities reported temperatures below the freezing threshold for vaccines [1] and a more recent study in India reports even higher incidents of freeze exposure, especially at peripheral facilities [4]. The fact that freeze exposure was less frequently identified as a problem in our study than heat exposure could be due to the fact that heat exposure is often more evident (e.g., when power outages occur), may affect greater quantities of vaccine, and can be signaled with vaccine vial monitors. Freeze exposure is less obvious and likely affects smaller quantities of vaccine (e.g., placed near ice in vaccine carriers or near cold spots in refrigerators). Vaccines can therefore freeze and thaw, and health workers may not visually inspect vaccines for evidence of freeze damage before use. Although the shake-test can detect vaccines that have been frozen [27], it is not often used. Based on the number of reported incidents of ice build-up in refrigerators and improper icepack conditioning, the likelihood of unrecognized freeze events is high.

Both equipment and vaccine product improvements can help to address the problem of inadvertent vaccine freeze exposure. Better temperature monitoring, better training, and improved refrigeration and transport containers that prevent freezing are definitely needed. WHO is working to advance new categories of prequalified cold-chain equipment with built-in freeze protection [28]; vaccine carriers are the first to meet this criterion. In addition, vaccines containing aluminum adjuvant (one of the main causes of freeze sensitivity) can be formulated to be freeze-stable using methodology that has been placed in the public domain [29], [30], a feature for which the majority (68%) of those involved in vaccine purchasing were willing to pay a suggested premium. The addition of the freeze-stability characteristic would be most cost-effective for vaccines that are in development, as it would have negligible impact on the development cost or price of these vaccines [2], [31].

## 5 Limitations

The study only surveyed a small number of individuals in a few countries and should not be considered a representative sample. The findings presented are designed to illuminate the opinions of stakeholders with a variety of diverse perspectives and priorities.

## 6 Conclusions

Immunization stakeholders in the participating low- and middle-income countries expressed interest in the availability and purchase of vaccine products with improved heat- and freeze-stability characteristics. Respondents also valued the concept of receiving vaccines whose labels reflect their ability to withstand short-term exposure to high temperatures. They recognized the benefits of such labeling in terms of improved information about potential vaccine damage should cold-chain breaks occur as well as in the possibility of purposefully removing vaccines from refrigeration as long as CTC conditions are maintained. Their concerns about the issues surrounding changes to vaccine handling conditions for a subgroup of EPI vaccines highlight the need to carefully consider purposeful removal of CTC-qualified vaccines from the cold chain to ensure that the benefits are maximized and outweigh the potential risks and additional efforts required.

## Figures and Tables

**Table 1:** Overview of study participants.

	Brazil	China	India	Peru	Philippines	Tanzania	Zanzibar^b^	Total
**Country information**								
Economic classification^a^	UMIC	UMIC	LMIC	UMIC	LMIC	LIC	LIC	
GAVI-eligible (at time of study)^d^	No	No	Yes	No	No	Yes	Yes	

**Distribution of participants by role (and supply chain level)**								
Decision-makers and advisors (national)	7	1	7	3	9	3	0	30
EPI managers (national)	1	0	1	1	1	1	1	6
Health and logistics personnel^c^								
National level	0	0	0	1	2	1	1	5
Region/province level	3	3	3	7	4	6	2	28
Municipal/district level	4	5	6	9	16	6	4	50
Facility level	4	2	7	6	13	4	3	39
**Total number of participants**	19	11	24	27	45	21	11	158

a Country classification according to World Bank categories: LIC: low-income country; LMIC: lower-middle-income country; UMIC: upper-middle-income country. Source: http://data.worldbank.org/country.

b Zanzibar is assumed to be treated as part of Tanzania in terms of GAVI-eligibility, although it has a separate EPI Manager and Ministry of Health team.

c GAVI eligibility is based on support for pentavalent vaccine. Source: http://www.gavialliance.org/results/countries-approved-for-support/.

d Health and logistics personnel include cold-chain managers, logisticians, and health workers.

## Footnotes

2 This article deals with a sub-set of the survey questions. Other results will be presented separately.

3 This represents a hypothetical scenario drafted prior to the establishment of WHO programmatic criteria for CTC-labeled vaccines. WHO criteria states “the vaccine must be able to tolerate ambient temperatures of at least +40 °C for a minimum of three days.” Source: World Health Organization. Vaccine Management and Logistics: Controlled Temperature Chain. October 9, 2015. Available at: http://www.who.int/immunization/programmes_systems/supply_chain/resources/tools/en/index6.html.
